# Comparison of a Novel Modality of Erbium-Doped Yttrium Aluminum Garnet Laser-Activated Irrigation and Ultrasonic Irrigation against Mature *Enterococcus faecalis* Biofilm—An In Vitro Study

**DOI:** 10.3390/bioengineering11100999

**Published:** 2024-10-04

**Authors:** Gabrijela Kapetanović Petričević, Antonio Perčinić, Ana Budimir, Anja Sesar, Ivica Anić, Ivona Bago

**Affiliations:** 1 Department of Endodontics and Restorative Dentistry, School of Dental Medicine, University of Zagreb, 10 000 Zagreb, Croatia; gkapetanovic@sfzg.hr (G.K.P.); anic@sfzg.hr (I.A.); 2 Department of Microbiology, University Hospital Center, 10 000 Zagreb, Croatia; antonio.percinic@kbc-zg.hr (A.P.); abudimir@kbc-zagreb.hr (A.B.); 3 Department of Endodontics, Oral Patology and Periodontology, Dental Polyclinic Zagreb, 10 000 Zagreb, Croatia; dr.anjasesar@gmail.com

**Keywords:** irrigation, *Enterococcus faecalis*, laser

## Abstract

In this in vitro study, we aimed to evaluate and compare the antibacterial efficacy of a novel erbium-doped yttrium aluminum garnet laser modality, shock wave enhanced emission of photoacoustic streaming (SWEEPS), ultrasonically activated irrigation (UAI), and single needle irrigation (SNI) against old bacterial biofilm. A two-week-old *Enterococcus faecalis* biofilm was cultivated on transversal dentinal discs made from the middle third of the roots of single-rooted, single-canal premolars. Biofilm growth was confirmed using scanning electron microscopy (SEM) and confocal laser scanning microscopy (CLSM). The dentine samples were randomly distributed into three experimental groups and one control group based on the irrigation protocol used: Group 1, SWEEPS; Group 2, UAI; and Group 3, SNI. The root canals were irrigated with a 3% sodium hypochlorite solution. Antibacterial efficacy was evaluated quantitatively through bacterial culture and qualitatively through CLSM and SEM. Both SWEEPS and UAI demonstrated a statistically significant reduction in *Enterococcus faecalis* colony-forming units (CFUs) (*p* < 0.001), while SNI did not show a statistically significant reduction (*p* = 0.553). No significant difference was observed between the efficacy of SWEEPS and UAI (*p* > 0.05). The SWEEPS and UAI techniques were equally effective in eliminating mature *E. faecalis* biofilm.

## 1. Introduction

Mature bacterial biofilm poses a major clinical challenge in root canal treatment because of its resistance to standard antimicrobial protocols and its ability to adapt to root canal environmental changes [1]. *Enterococcus faecalis* (*E. faecalis*) is a known, highly resistant bacterium with specific characteristics, such as its capacity to withstand sodium hypochlorite (NaOCl) concentrations as high as 6.5% [2] and pH regulation [3], induce hydroxyapatite reprecipitation in mature biofilms, and form calcified monobiofilms [4]. Clinical studies have shown that it is more frequently associated with secondary and persistent endodontic infections [5,6]. The success rate of root canal retreatment varies between 45% and 83% [7,8], which is significantly lower than that for initial root canal treatments. The eradication of *E. faecalis* has been of immense interest in the last decade [9]. Disintegration and elimination of bacterial biofilm could be improved by mechanically using different activated irrigation techniques, including ultrasonically activated irrigation (UAI) and novel laser-activated irrigation (LAI) techniques [10,11]. Since *E. faecalis* can invade dentinal tubules to depths of approximately 500 μm [12], ensuring deeper irrigant penetration and increasing cleaning efficiency within the three-dimensional root canal system is important [12].

UAI energizes the irrigant solution within the canal, creating acoustic streaming, which improves biofilm removal [13]. In contrast, LAI is based on vapor bubble dynamics produced by pulsed lasers, creating specific bubble and cavitation phenomena and acoustic streaming [14]. Previous studies reported that LAI has an effective bactericidal activity and improves smear layer elimination, even from the apical third of the root [15,16]. A novel LAI technique, shock wave enhanced emission photodynamic streaming (SWEEPS), uses a dual-pulse modality, where the second laser pulse is applied just before the collapse of the first laser pulse’s bubble, amplifying the cavitation effect [17,18]. The AutoSWEEPS modality, which uses ultrashort pulses (25 µs) with minimal energy levels (20 mJ), has been reported to be approximately 50% more effective than the standard single-pulse modality in generating pressures within the root canal and significantly enhancing irrigant penetration into the dentinal tubules [19]. Furthermore, recent studies confirm SWEEPS efficacy in the removal of smear layers and debris [11], as well as residual root canal filling material [20,21]. Recent studies confirmed SWEEPS efficacy in *E. faecalis* biofilm removal when combined with antimicrobial photodynamic therapy (aPDT) [22,23] because it can effectively increase the photosensitizer distribution in the root canal system. However, there is a lack of studies comparing SWEEPS with other activated irrigation techniques. To our best knowledge, only a few recent studies have compared SWEEPS with other irrigation methods for *E. faecalis* biofilm removal [24]; two studies on multispecies biofilm removal [25,26] reporting similar antimicrobial effects across tested groups.

The aim of this in vitro study was to evaluate and compare the antibacterial efficacy of SWEEPS, UAI, and single needle irrigation (SNI) against a two-week-old *E. faecalis* monobiofilm. The null hypothesis was that there was no significant difference in the eradication of mature *E. faecalis* biofilm between the SWEEPS, UAI, and SNI.

## 2. Materials and Methods

### 2.1. Selection and Preparation of Dentine Discs

Based on a power analysis using repeated measures analysis of variance (RM-ANOVA), with a significance level (alpha) of 0.05 and 90% test strength, it was necessary to include at least 11 samples per group in the research.

For the purpose of this study, extracted human single-rooted, single-canal premolars were collected. The exclusion criteria included previous root canal treatment and the presence of carious lesions. The presence of a single round root canal was confirmed through a cone beam computed tomography (CBCT) scan (Cranex 3DX; Soredex, Tuusula, Finland; parameters: FOV 595 (5.0 mm) mm; ENDO, 85 µm; 6.3 mA; 90 kV; 8.7 s; i 450.3 mGycm^2^).

Transversal dentinal discs (1 mm thick, 5 × 5 mm) were made from the middle third of the root using a diamond disk (Isomet 1000, Buehler, IL, USA). Both sides of the samples were polished using three polishing discs with varying degrees of fineness (Presi France, P320, P600, P1200). The samples were then sterilized in hot plasma (Sterrad 100 NX, ASP, Schaffhausen, Switzerland).

### 2.2. Enterococcus faecalis Biofilm Formation

The *E. faecalis* ATCC strain (ATCC 29212, AmericanType Culture Collection, Manassas, VA, USA) was used in this study to form a two-week-old bacterial biofilm. The bacterial suspension density was adjusted to 0.5 McFarland, as measured via a densitometer (Densimat, Bio Mérieux, Marcy l’Etoile, France). Sterile dentine discs were placed in 1 mL microtiter wells (Thermo Fisher Scientific, Waltham, MA, USA) containing a Brain Hearth Infusion (BHI) broth (Sigma Aldrich, Burlington, MA, USA). The BHI was replaced every 48 h for 14 days.

### 2.3. Experimental Irrigation Protocols

A total of 72 dentine samples were prepared and inoculated with bacterial biofilm. In each experimental group, 13 samples (n = 13) were used for quantitative microbiological analysis, while four samples were used for scanning electron microscopy (SEM) and confocal laser microscopy (CFLM) analysis.

In all three experimental groups, each dentine sample was placed into a 1 mL microtiter well (Thermo Fisher Scientific, Massachusetts, USA) containing 500 μL of 3% NaOCl solution. The samples were then subjected to a 90 s irrigation protocol, divided into three cycles of 30 s each use. For each cycle, the dentine sample was transferred to a new well (Thermo Fisher Scientific, Massachusetts, USA) containing 500 μL of fresh 3% NaOCl solution.

#### 2.3.1. Group 1: SWEEPS

The samples were treated using an erbium-doped yttrium aluminum garnet (Er: YAG) laser (LightWalker AT, Fotona, Ljubljana, Slovenia) equipped with a radial laser tip (600 µm, 9 mm, Fotona, Ljubljana, Slovenia). During activation, the radial laser tip was inserted into the well and maintained in a fixed position. The laser parameters were set at Auto SWEEPS mode: pulse energy (20 mJ), pulse frequency (15 Hz), average power (0.60 W), pulse duration (25 µs), and peak power (800 W).

#### 2.3.2. Group 2: Ultrasonically Activated Irrigation

The samples were activated using an endodontic ultrasonic tip (Irri S, 25/25, VDW, Munich, Germany) connected to an ultrasound device (VDW ULTRA; VDW, Munich, Germany), which was set at 20% power as required for the irrigation mode by the manufacturer. During activation, the ultrasonic tip was placed stationary inside the microtiter well.

#### 2.3.3. Group 3: Passive Irrigation (PI)

The samples were passively immersed in microtiter wells filled with 500 μL of 3% NaOCl. As previously explained, after each 30 s cycle, the discs were transferred into another well filled with fresh 3% NaOCl.

Following the irrigation protocol, each sample was immersed in a well containing a sodium thiosulfate solution for 30 s to neutralize NaOCl after the termination of the experimental irrigation protocol. Finally, each sample was immersed in sterile saline.

For each experimental group, seven additional dentine samples were used as controls, receiving no treatment. Five of these control samples served for quantitative analysis to determine the initial number of *E. faecalis* CFUs, while the other two were for SEM and CLSM analyses to confirm the initial presence of *E. faecalis* bacterial biofilm.

### 2.4. Culture Method and Quantification of E. faecalis Colony Forming Units

Quantification of bacteria colony forming units (CFUs) was performed using the culture method, before the irrigation protocols (controls) and after the irrigation protocols. Each dentine sample was placed in a sterile 1 mL tube containing 500 μL of sterile saline and vortexed (V-1 plus, Biosan, Riga, Latvia) for 30 s in touch mode. The prepared suspension was then serially diluted, and 20 µL were added to wells containing 180 μL Mueller–Hinton broth (Sigma-Aldrich, Burlington, MA, USA). Following the serial dilutions, 10 μL of each suspension were plated onto blood agar plates (211037, Becton Dickinson, New York, NY, USA), starting from the highest dilution to the lowest. The samples were incubated for 24 h in a chamber at 37 °C. After 24 h, the colonies were counted, and the specific number of *E. faecalis* CFU was calculated based on the degree of dilution. The reduction in *E. faecalis* CFUs following treatment was evaluated by comparing them with the controls.

### 2.5. Scanning Electron Microscopy Analysis

SEM analysis was performed before the irrigation protocols (controls) and after the irrigation protocols. After fixation in formalin, the dentine samples were dehydrated in increasing concentrations of alcohol: 55%, 60%, 75%, 80%, and 95%. Afterward, the samples were exposed to drying at the critical point where water was replaced with CO_2_ under a pressure of 80 bar. SEM analysis was conducted on a field emission SEM (FE-SEM) model JSM-7000F (Jeol Ltd., Tokyo, Japan). Randomly selected surfaces on the samples were examined, and four micrographs per specimen were captured at magnifications of 5000× and 10,000×, with a 1-micrometer thickness. The analysis was performed by an individual who was blinded to the preceding sample irrigation treatment.

### 2.6. Confocal Laser Scanning Microscopy Analysis

CLSM analysis was performed before the irrigation protocols (controls) and after the irrigation protocols. The dentine discs were stained with the LIVE/DEAD kit (Invitrogen LIVE/DEAD BacLight Bacterial Viability kit), which contains two dyes, SYTO 9 (green fluorescence) and propidium iodide (red color).

The samples were then covered with 10 µL staining solution consisting of 1 mL 0.9% NaCl, 1 µL SYTO 9, and 1 µL propidium iodide and incubated for 15 min in the dark at 25 °C. After staining, the samples were gently irrigated with saline. The green dye binds to the cell wall of living bacteria, while the red dye binds to the damaged bacteria cell wall. Consequently, the samples were analyzed using a confocal laser microscope (Leica TSC SP8; Leica Microsystems GmbH, Wetzlar, Germany). Randomly chosen surfaces on the sample were taken into consideration, and four micrographs per specimen were taken. The analysis was performed by an individual who was blinded to the preceding sample treatment.

Researchers who performed culture method and CFUs quantification, SEM and CFLM, did not know whether the samples were taken from an experimental group or a control group.

### 2.7. Statistical Analysis

To distribute results and compare the number of *E. faecalis* CFUs pre- and post-treatment, the non-parametric Mann–Whitney U test was used because of data inhomogeneity (*p* < 0.05). A significance level of *p* = 0.05 was used for all statistical tests. The data analysis was performed using the IBM Statistics 19.0.0.1 software package. (StatSoft, Tulsa, OK, USA).

## 3. Results

### 3.1. Results of the Culture Method

Table 1 shows the number of *E. faecalis* CFUs for each tested group (SWEEPS, UAI, PI) and control samples in each group.

In intragroup analysis, both SWEEPS and UAI showed a statistically significant reduction in *E. faecalis* CFUs (*p* < 0.001), while the PI group did not demonstrate a statistically significant reduction (*p* = 0.553) (Table 1).

Intergroup analysis revealed no statistically significant difference between the SWEEPS and UAI groups (*p* > 0.05).

### 3.2. Qualitative Results of Scanning Electron Microscopy and Confocal Laser Scanning Microscopy

**Figure 1:**
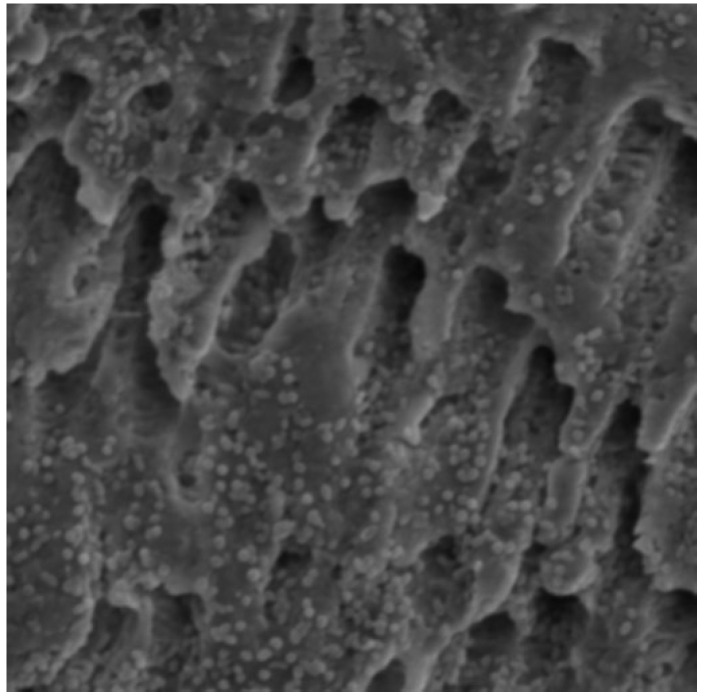
Scanning electron microscopy of *Enterococcus faecalis* biofilm on dentine disc after two weeks of incubation.

**Figure 2:**
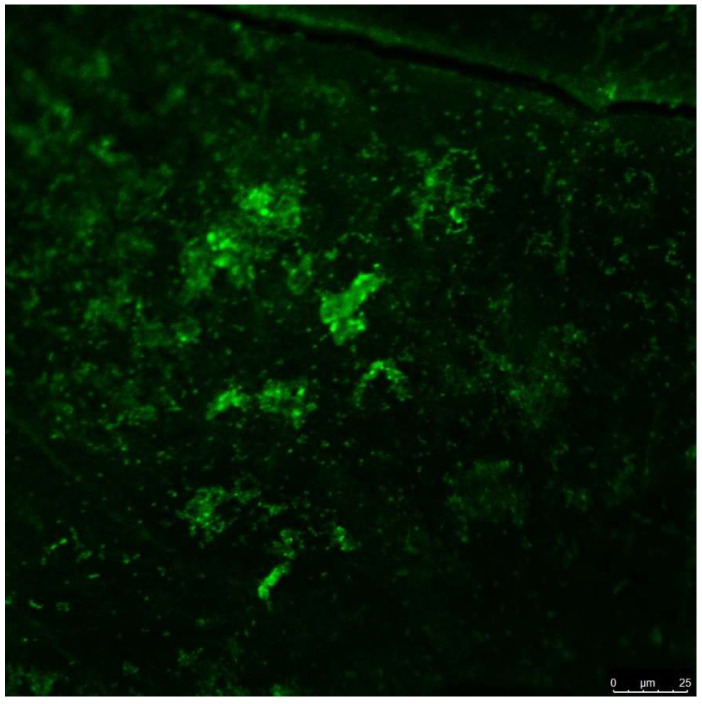
Confocal laser scanning microscopy of *Enterococcus faecalis* biofilm on dentine discs after two weeks of incubation.

Figure 1 (SEM) and Figure 2 (CLSM) illustrate the diffuse growth of *E. faecalis* biofilm on dentine discs after two weeks of incubation.

**Figure 3:**
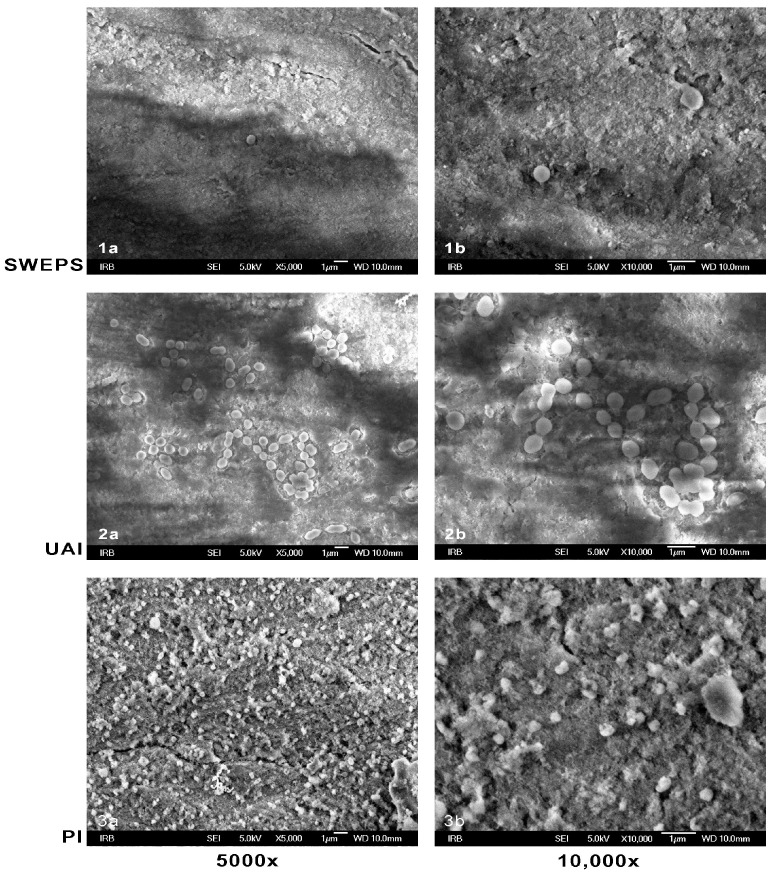
Scanning electron microscopy of representative dentine discs with *Enterococcus faecalis* (*E. faecalis*) biofilm under 5000× (**a**) and 10,000× (**b**) magnification after the irrigation protocols: (**1a**,**1b**). SWEEPS: visible small number of isolated planktonic cells of *E. faecalis*; (**2a**,**2b**). Ultrasonically activated irrigation (UAI): few *E. faeacalis* colonies grouping into strains and pairs; (**3a**,**3b**). Passive irrigation (PI): dense *E. faeacalis* colonies diffusely spread all over the sample’s surface.

Figure 3 shows the SEM of dentine discs following the irrigation protocols under 5000× and 10,000× magnification. In the SWEEPS group (Figure 3(1a,1b)), a small number of planktonic *E. faecalis* cells were visible with great areas of clean dentine surface. In the UAI group (Figure 3(2a,2b)), fewer *E. faecalis* colonies were present, mostly clustering into strains and pairs. In contrast, the PI group (Figure 3(3a,3b)) exhibited diffusely spread *E. faecalis* colonies across the dentine surface, confirming the inadequate efficiency of the PI based on SEM analysis.

**Figure 4:**
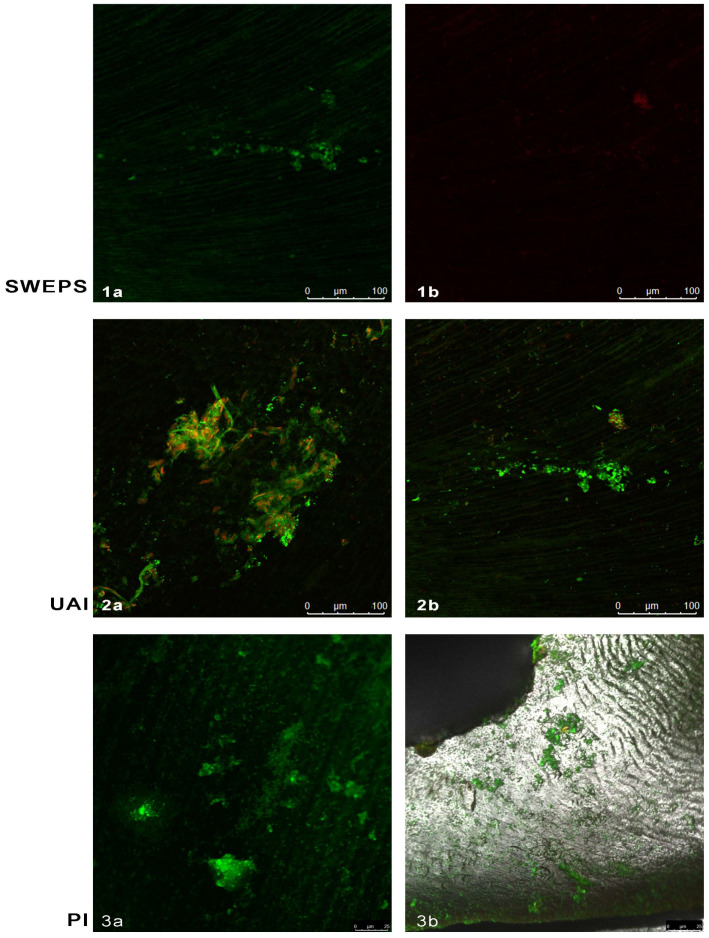
Confocal laser scanning microscopy of representative dentine discs with *Enterococcus faecalis* (*E. faecalis*) biofilm after the irrigation protocols: (**1a**,**1b**). SWEEPS: a few areas adhered to living *E. faecalis* colonies (green) are observed, as well as several areas where dead bacteria accumulated (red) (**b**) and a large area of clean dentin; (**2a**,**2b**). Ultrasonically activated irrigation (UAI): areas with dead colonies (red) of *E. faecalis*, and significant areas of live bacteria colonies still (green); (**3a**,**3b**). Passive irrigation (PI): large areas of live *E. faecalis* bacteria colonies (green).

Figure 4 shows the CFLM of dentine discs following the irrigation protocols. In the SWEEPS group (Figure 4(1a,1b)), large areas of clean dentine were visible, with few areas with viable *E. faecalis* colonies (green) (Figure 4(1a)), while several areas contained accumulated dead bacteria (red) (Figure 4(1b)). In the UAI group, an increase in the density of dead *E. faecalis* colonies (red) was visible, and a lower density of viable colonies (green) compared with the control sample surfaces (Figure 4(2a,2b)). However, colonies were still attached to the sample surface in significant numbers. In the PI group, a large number of viable *E. faecalis* bacteria clusters (green) were present, with a minimal number of dead bacterial cells (red) (Figure 4(3a,3b)).

## 4. Discussion

The results of this study revealed that both evaluated activated irrigation techniques, SWEEPS and UAI, were more effective than PI in removing bacterial biofilm from the dentinal surface under in vitro conditions. Furthermore, no significant difference was found between SWEEPS and UAI. Thus, the null hypothesis can be partially rejected. Over the last decade, numerous studies have highlighted LAI as a method of choice for cleaning complex canal anatomies [27], dissolving pulp tissue [28], and removing smear layers and hard tissue debris [29]. Lei et al. [30] demonstrated that LAI increased the efficiency of lower NaOCl concentrations and equalized NaOCl efficiency across different concentrations (0.5–5.25%) in eradicating *E. faecalis* biofilm. In this study, the NaOCl concentration was consistent across all groups (3%), and the SWEEPS technique showed significantly greater biofilm reduction than PI, as confirmed by SEM and CLSM analyses (Figure 3 and Figure 4). Recently, CLSM has been recommended as a powerful technique for evaluating endodontic disinfection [31].

The results of this study align with those of a recently published study by Robberecht et al. [25], who also found similar efficacy between SWEEPS and sonic irrigation, with needle irrigation being the least effective against biofilm-mimicking hydrogel in the isthmus region of 3D-printed models. Furthermore, in a more recent study by Akdere et al. [24], SWEEPS and UAI exhibited equal antimicrobial efficacy against *E. faecalis* biofilm in internal resorption cavities. No significant difference was observed between UAI and SWEEPS in this study; however, SWEEPS provided complete elimination of bacteria in three samples. These results in comparison with the study by Akdere et al. [24] suggest that, in a simple canal anatomy, the SWEEPS technique does not provide extra benefit compared to the UAI. More complex root canal anatomy requires advanced irrigation techniques. The results of this study suggest the probable greater potential of the SWEEPS technique; however, more studies on the topic are required. Moreover, SEM and CLSM analyses revealed cleaner dentine disc surfaces after SWEEPS, with few bacteria cells present, compared to UAI, which still contained groups of *E. faecalis* cells (Figure 3 and Figure 4). In contrast to our findings, De Meyer et al. [26] reported greater efficacy of Er: YAG LAI compared to UAI against dual-species biofilm in a resin root canal model. However, De Meyer et al. used a different biofilm model [26] and employed saline solution as an irrigant, whereas in this study, 3% NaOCl was used. Possibly, the high efficacy of NaOCl could have compensated for the lower efficacy of UAI in this study. Bao et al. [32] evaluated in their study the efficacy of photon-induced photoacoustic streaming (PIPS) and SWEEPS against multispecies biofilms within apical artificial grooves and dentinal tubules, compared to conventional needle irrigation (CNI), UAI, and Eddy. They found SWEEPS, PIPS, and Eddy had similar antibacterial efficacy but greater efficacy than CNI and UAI.

The powerful action of the AutoSWEEPS mode is because of dual pulse modality, meaning the temporal separation between the pulse pairs is continuously swept back and forth, providing an adequate cavitation effect since oscillation times of cavitation bubbles vary considerably depending on pulse energy and cavity diameter [27]. Since oscillation times are shorter in larger cavities [33], leading to more effective bubble dynamics, the large reservoir used (1 mL wells) could also be the reason why, in some samples, biofilm was completely detached. Swimberghe et al. [17] explained the mechanism between biofilm detachment from root canal walls during LAI and the mechanical disruption of fragments coronally. They emphasized that the oscillation of small cavitation bubbles was a determinant in the generation of rapid vertical irrigant movement, causing strong streaming and biofilm detachment.

A few limitations to this study should be emphasized. First, the biofilm in this study was grown on dentinal discs, making it more accessible to the activation technique than the biofilm in complex root canals. Furthermore, the collapse of the bubble, which generates shock waves during SWEEPS, may have occurred easily in the large liquid reservoir used in this study. In confined spaces such as root canals, friction slows down bubble dynamics, leading to shock wave reduction or complete absence [25]. However, Swimberghe et al. [17] in their study used a high-speed camera to capture biofilm detachment and its coronal displacement in the root canal using single-pulse LAI, proving its efficacy in the root canal. They explained that LAI in root canals is based on the generation of smaller cavitation bubbles throughout the entire canal, distant from the primary bubble during its implosion. Moreover, a recent study by Bao et al. [33] confirmed that SWEEPS outperformed needle irrigation, EDDY, UAI, and LAI with photon-induced photoacoustic streaming in treating multispecies biofilms within apical root canals and dentinal tubules. The stationary reservoir of the irrigant could also be the reason of the significantly reduced efficacy of only passive disinfection, in which the discs were only placed in the well filled with NaOCL. Future studies should evaluate the efficacy of SWEEPS compared to standard activation and passive irrigation techniques in more complex root canal systems. Clinical studies with the primary outcome of the SWEEPS in the healing of periapical lesions are also needed.

## 5. Conclusions

Within the limitations of this study, the SWEEPS and UAI techniques were equally effective in eliminating mature *E. faecalis* biofilm from dentinal discs and demonstrated superiority over SNI. However, since some samples were completely free of biofilm only in the SWEEPS group, its potential advantage over other techniques warrants further investigation.

## Figures and Tables

**Table 1:** Number of *E. faecalis* colony forming units (CFUs) in each tested and control group after the experimental irrigation protocol.

			Minimum	Maximum	Percentile 25	Median	Percentile 75
Number of CFUs	SWEEPS	Control	5 × 10^12^	3.5 × 10^13^	5 × 10^12^	1 × 10^13^	1.5 × 10^13^
		Experimental group	0	5 × 10^4^	0	0	0
	UAI	Control	2 × 10^13^	1 × 10^14^	2.5 × 10^13^	7 × 10^13^	7.5 × 10^13^
		Experimental group	5 × 10^5^	2 × 10^9^	5 × 10^6^	5 × 10^6^	5 × 10^8^
	PI	Control	5 × 10^7^	2.5 × 10^13^	5 × 10^8^	5 × 10^8^	1 × 10^10^
		Experimental group	1 × 10^5^	2.5 × 10^13^	5 × 10^6^	5 × 10^8^	5 × 10^11^

SWEEPS, Shock Wave Enhanced Emission Photoacoustic Streaming; UAI, ultrasonically activated irrigation; PI, passive irrigation.

## Data Availability

Data is contained within the article.
